# Psychometric evaluation of the DAILY EATS questionnaire in individuals living with obesity

**DOI:** 10.1186/s41687-020-00259-w

**Published:** 2020-11-23

**Authors:** John Fastenau, Heather Rozjabek, Shanshan Qin, Lori McLeod, Lauren Nelson, Jia Ma, Nimanee Harris

**Affiliations:** 1 Janssen, 700 US Highway Route 202, Raritan, NJ 08869 USA; 2 RTI Health Solutions, 3040 East Cornwallis Road, Post Office Box 12194, Research Triangle Park, NC 27709-2194 USA

**Keywords:** Obesity, Patient-reported, Hunger, Appetite, Qualitative

## Abstract

**Background:**

Physiological and behavioral factors including hunger, satiety, food intake, and cravings are health determinants contributing to obesity. Patient-reported outcome (PRO) measures focused on eating-related factors provide insight into the relationships between food choice and quantity, weight change, and weight-loss treatment for individuals living with obesity. The DAILY EATS is a novel 5-item, patient-reported measure evaluating key eating-related factors (Worst and Average Hunger, Appetite, Cravings, and Satiety).

**Methods:**

Psychometric analyses, consistent with regulatory standards, were conducted to evaluate the DAILY EATS using data from two randomized trials that included individuals with severe obesity without diabetes (NCT03486392) and with severe obesity and type 2 diabetes (NCT03586830). Additional measures included Patient Global Impression of Status (PGIS) and Patient Global Impression of Change items, Impact of Weight on Quality of Life-Lite, Ease of Weight Management, and Patient-Reported Outcomes Measurement Information System Physical Function Short Form 8b and 10a. The reliability, validity, and responsiveness of the DAILY EATS were assessed, and a scoring algorithm and thresholds to interpret meaningful score changes were developed.

**Results:**

Item-level analyses of the DAILY EATS supported computation of an Eating Drivers Index (EDI), comprising the related items Worst Hunger, Appetite, and Cravings. Internal consistency (Cronbach’s coefficient alphas ≥0.80) and test-retest reliability (coefficients > 0.7) of the EDI were robust. Construct validity correlation patterns with other PRO measures were as hypothesized, with moderate to strong significant correlations between the EDI and PGIS-Hunger (0.30 ≤ r ≤ 0.68), PGIS-Cravings (0.33 ≤ r ≤ 0.77) and PGIS-Appetite (0.52 ≤ r ≤ 0.77). Anchor- and distribution-based analyses support reductions ranging from 1.6 to 2.1 as responder thresholds for the EDI, representing meaningful within-person improvement.

**Conclusions:**

The DAILY EATS individual items and the composite EDI are reliable, sensitive, and valid in evaluating the concepts of hunger, appetite, and cravings for use in individuals with severe obesity with or without type 2 diabetes.

## Background

A recent initiative to develop a patient-centered disease-illness model for obesity identified physiological and behavioral factors, including hunger, satiety, food intake, and cravings, as health determinants contributing to obesity [1]. Specifically, potential eating-related barriers to weight loss included difficulties in controlling hunger and appetite and the lack of the sensation of fullness after eating a meal. Individuals living with obesity may be able to lose weight or maintain a healthier weight if they have more control over these eating-related factors, thus allowing for more appropriate meal portion-sizes and fewer cravings, particularly for foods high in calories. For individuals living with obesity, with or without concomitant type 2 diabetes mellitus (T2DM), weight loss may relieve physical, social, emotional, and functional impacts associated with obesity [2, 3].

While select weight-loss medications target hormones that control hunger and satiety, patients’ interpretations of such concepts and their role in chronic weight management are not well understood. Patient-reported outcome (PRO) measures focused on factors related to eating may help facilitate better understanding of the relationships between eating-related factors, weight change, and weight-loss treatment.

The DAILY EATS: Measuring Daily Eating Factors questionnaire is a novel 5-item, patient-reported measure developed to provide information about the key factors associated with eating and includes assessments of hunger (2 items), appetite, cravings for unhealthy food, and satiety after meals. An 11-point numerical rating scale (0–10) is used for each item, with a higher value indicating more hunger, bigger appetite, stronger cravings, or greater satiety. Selection of potential concepts for the DAILY EATS was informed by the results of previously conducted obesity-related research, input of clinical and PRO experts, and qualitative research that included concept elicitation interviews with 35 overweight or obese individuals, either with or without T2DM [4]. The results of the qualitative research informed the development of a conceptual model of the hypothesized relationships among the eating-related factors, as well as the impacts of these factors on food quantity and choice, identified as important to patients during the concept elicitation interviews (see Fig. 2 in Appendix).

The pilot version of the DAILY EATS, initially referred to as the Eating-Related Concepts Questionnaire, was subsequently debriefed in three rounds of interviews and refined between each round, as needed, based on participant feedback. The DAILY EATS is designed to be completed as a daily diary (24-h recall period) at the same time each day, preferably in the evening. The daily responses are used to compute item-level weekly averages.

The objectives of this research were to conduct a psychometric evaluation of the DAILY EATS, assessing its reliability, validity, and responsiveness, as well as assess structure to develop optimal composite scores and an interpretation guideline. The psychometric evaluation was conducted by using data from two phase 2 clinical trials of a novel weight-loss medication: one conducted in individuals with severe obesity (body mass index [BMI], 35–50 kg/m2) and without diabetes (Study 1; NCT03486392) and a separate study conducted in individuals with severe obesity and T2DM (Study 2; NCT03586830). Development and psychometric evaluation of the DAILY EATS were conducted in a manner consistent with the review criteria described in the United States Food and Drug Administration’s Patient-Reported Outcome Guidance [5]. Additional details about the psychometric evaluation are summarized in the Appendix.

## Methods

### Study measures

Instruments used in the psychometric analysis included the DAILY EATS diary; Patient Global Impression of Severity (PGIS) items related to hunger, appetite, cravings, satiety, and physical functioning; Patient Global Impression of Change (PGIC) items related to hunger, cravings, and physical functioning; the Impact of Weight on Quality of Life-Lite (IWQOL-Lite) measure; the single-item Ease of Weight Management (EWM) measure; and the Patient-Reported Outcomes Measurement Information System Physical Function Short Form (PROMIS PF SF) 8b and 10a measures. All instruments were administered on paper in Study 1 and Study 2. Higher scores for the DAILY EATS, PGIS items, and PGIC items are indicative of higher levels of hunger, appetite, cravings, and other eating-related behaviors, while higher scores for the IWQOL-Lite, EWM and PROMIS PF SF indicate better health-related quality of life, greater ease of weight loss, and physical functioning, respectively. Details related to the recall period and time points for the key measures, including the DAILY EATS, used in the psychometric evaluation are provided in Table 7 in Appendix.

### Study design and population

Data from the two studies were used separately to evaluate the psychometric properties of the DAILY EATS in individuals living with severe obesity. Study 1 was a randomized, phase 2b, double-blind, placebo-controlled and open-label active-controlled, parallel-group, multicenter, dose-ranging study to evaluate the safety and efficacy of a novel weight-loss medication in individuals with severe obesity without diabetes across 26 weeks of treatment. Study 2 was a randomized, phase 2b, double-blind, placebo-controlled, parallel-group, multicenter, dose-ranging study to evaluate the safety and efficacy of the same novel weight-loss medication in individuals with severe obesity with T2DM across 12 weeks of treatment. All psychometric analyses were conducted without reference to treatment group (i.e., data were pooled across treatment arms into a study-related analysis population). For each study, analyses were conducted using all patients in the modified intent-to-treat clinical analysis data set who completed at least one DAILY EATS item at least 1 day at baseline and also at least 1 day in a follow-up week. Both studies complied with the Declaration of Helsinki and were approved by the relevant investigational review boards or ethics committees for the respective study sites.

### Descriptive statistics, missing data, and DAILY EATS structure

The study populations and descriptive statistics for the supporting measures were summarized descriptively. Weekly average and change-score standard descriptive statistics were reported. Floor or ceiling effects for DAILY EATS items were defined as more than 18% of patients (approximately twice the expected probability for each of the 11 categories in a uniform distribution) selecting an extreme response category (e.g., 0 [Not hungry at all], 10 [Extremely hungry]).

The impact of missing DAILY EATS data was evaluated at the daily level of baseline to inform scoring rules for weekly averages using a missing data simulation: different subsets of daily responses to each item were deleted to assess the stability of the resulting distribution.

The pattern of inter-item correlations was evaluated at baseline and end of treatment (EOT: Week 26 in Study 1, Week 12 in Study 2) to inform potential DAILY EATS composite scores, such that moderate correlations (*r* ≥ 0.30) supported composite formation and strong correlations *r* > 0.80 indicated potential redundancy. Exploratory factor analysis (EFA) was conducted using an inter-item Pearson correlation matrix based on the weekly scores at baseline and maximum likelihood estimation with robust standard errors. The size of the eigenvalues [6] and the scree plot [7] guided the decision regarding dimensionality.

Each psychometric property was evaluated for the DAILY EATS weekly items and, after reviewing the item-level and the scoring analyses, a single composite of three DAILY EATS items, the Eating Drivers Index (EDI), was developed for scoring purposes. The EDI is scored as the average of the weekly scores for Worst Hunger (Item 2), Appetite (Item 3), and Cravings (Item 4). Early qualitative research [4] and the item-level quantitative analyses support the relevance of all five DAILY EATS items. Future studies may consider reporting both the EDI and the five individual items scores. For the purposes of this manuscript, the remaining properties focus primarily on the EDI composite and the three component DAILY EATS items of Worst Hunger, Appetite, and Cravings.

### Reliability

Internal consistency reliability analyses evaluated the degree to which items were associated with one another. Cronbach’s coefficient alpha [8] was computed at baseline and EOT. The approximate range of optimal alphas suggested by Streiner and Norman [9] is between 0.70 and 0.90, indicating a set of items that is strongly related and capable of supporting a unidimensional scoring structure but not redundant.

The test-retest reliability of the DAILY EATS weekly item scores was assessed by computing intraclass correlation coefficients (ICCs) among patients considered to be stable based on an external criterion over the test-retest period. A two-way mixed-effects analysis of variance (ANOVA) with absolute agreement for single measures was used to compute test-retest reliability ICCs [10, 11]. Study 1 data used Week 15 (test) and Week 26/EOT (retest) for a subgroup with no corresponding PGIS change. Study 2 data used baseline (test) and Week 12/EOT (retest) for a subgroup with no corresponding PGIS change.

### Validity

#### Construct validity

Construct validity describes the relationships among multiple indicators of a construct and the degree to which they follow predictable patterns. Cross-sectional correlations were computed between weekly DAILY EATS item and EDI composite scores and supporting measures (i.e., PGIS item scores, EWM, BMI, IWQOL-Lite domain and total scores, and PROMIS PF SF 8a and 10b total scores) at baseline and EOT. The magnitude and direction of the resulting correlation coefficients were compared with respect to specific a priori hypotheses and to Cohen’s guideline [12] for interpreting correlation coefficients: absolute values of correlations of 0.50 or greater are considered strong, correlations that fall between 0.30 and 0.49 are moderate, and those that fall between 0.10 and 0.29 are small. Moderate to strong correlations were hypothesized for the weekly DAILY EATS item scores and the EDI composite with corresponding PGIS items (e.g., between DAILY EATS Worst Hunger items and PGIS-Hunger item), whereas smaller correlations were hypothesized between DAILY EATS items and the EDI composite with the PGIS-Physical Functioning (PGIS-PF) item. Trivial (|r| <  0.1) to small correlations were hypothesized for DAILY EATS item scores and the EDI composite with physical function scores based on the PROMIS PF SF 8b and SF 10a.

#### Known-groups validity

Known-groups analyses comparing subgroups of interest were conducted to evaluate the discriminating ability of the DAILY EATS weekly item and EDI composite scores at baseline and EOT. Analyses of variance, with the use of overall F test and pairwise comparisons based on a priori hypotheses, were conducted to examine mean differences in weekly DAILY EATS item and EDI composite scores between patients classified into subgroups based on the corresponding PGIS items. It was hypothesized that individual DAILY EATS item and EDI composite scores would differentiate between patients who report low levels of eating-related issues versus those who report higher levels on the corresponding PGIS items. It also was hypothesized that patients who reported little to no difficulty with their weight management on the EWM would have lower DAILY EATS item and EDI composite scores, on average, than those patients who report higher levels of difficulty in managing weight.

### Responsiveness

The DAILY EATS’ responsiveness—or its ability to detect change when change is expected—was evaluated using multiple methods: by computing correlations of change from baseline to EOT in the weekly DAILY EATS item and EDI composite scores and the supporting outcome measures, ANOVA, and effect-size estimates of change. Specifically, longitudinal correlations were computed between changes in weekly DAILY EATS item and EDI composite scores and changes in the supporting measures (i.e., corresponding PGIC items, weight change percentage, and changes in corresponding PGIS items, EWM, BMI, IWQOL-Lite, and PROMIS PF SF 8b and SF 10a) at the EOT. For the ANOVAs (using overall F test, pairwise comparisons, and effect sizes), it was hypothesized that patients who had improved scores on the corresponding PGIS (or PGIC) would have larger changes indicative of improvement than would patients who have remained the same or worsened on these assessments. For correlational (Pearson) analyses, the following correlations were hypothesized: (1) Moderate to strong correlations between changes in weekly DAILY EATS item and EDI composite scores and changes in the corresponding PGIS items; smaller correlations between changes in weekly DAILY EATS item and EDI composite scores and the change in PGIS-PF; (2) Moderate to strong correlations between changes in DAILY EATS Worst Hunger and Appetite items and PGIC-Hunger; moderate to strong correlations between the change in the weekly DAILY EATS Craving item and PGIC-Craving; smaller correlations between the changes in weekly DAILY EATS item and EDI composite scores and PGIC-Physical Functioning (PGIC-PF); (3) Small correlations between changes in weekly DAILY EATS item and EDI composite scores and changes in the physical function scores from the IWQOL-Lite and the PROMIS PF SF 8b and 10a; and (4) Small to moderate correlations between changes in weekly DAILY EATS item and EDI composite scores and the weight change percentage. Effect sizes of approximately 0.20 were interpreted to represent small effects, those of approximately 0.50 represented moderate effects, and those greater than approximately 0.80 represented large effects [13].

### Interpretation of change

To identify patients who experienced a meaningful change, a threshold or responder definition was estimated for three weekly DAILY EATS items (Worst Hunger, Appetite and Cravings) and the EDI composite score. Both anchor-based and distribution-based methods were used to estimate thresholds defining meaningful within-person change, or responder definitions, of the weekly DAILY EATS item and EDI composite scores in individuals with severe obesity without diabetes (Study 1) and with T2DM (Study 2). An anchor-based approach is the primary method recommended in the PRO guidance [5] to define this threshold. Prior to applying anchor-based methods, the appropriateness of the anchor measures was assessed by reviewing responsiveness correlations. A commonly applied criterion for identifying an appropriate anchor measure was used: the magnitude of the correlation of change was required to be at least 0.371, based on achieving a large effect size using Cohen’s rule of thumb [14–17]. In addition, the size and direction of the mean and median change in the weekly DAILY EATS item and EDI composite scores by the change in the corresponding anchor measures were reviewed to confirm that greater improvement or worsening in the weekly DAILY EATS item and EDI composite scores was achieved by patients who showed greater levels of improvement or worsening on the change in the anchor measures.

A 1-point improvement on the related PGIS was selected a priori as the primary anchor. Distribution-based estimates were also conducted to provide additional information and to serve as secondary threshold estimates. Finally, to support the anchor-based methods, cumulative distribution function (CDF) and probability density function (PDF) plots were developed.

## Results

### Sample characteristics

Table 1 presents key baseline characteristics of the 99 patients from Study 1 (individuals with severe obesity without diabetes) and the 146 patients from Study 2 (individuals with severe obesity with T2DM) in the psychometric analysis sample. Patients without diabetes and those with diabetes had an average BMI of 40.9 and 40.3, respectively, and were aged, on average, 48.2 years and 56.4 years at time of study entry. Both samples contained a higher proportion of female patients (71.7%, 58.9%) than male patients (28.3%, 41.1%), and patients were predominantly white (79.8%, 69.2%) and of non-Hispanic or Latino ethnicity (79.8%, 74.4%).

**Table 1:** Patient Characteristics at Baseline (Psychometric Analysis Sample)

Patient characteristic	STUDY 1 Severely obese without diabetes (*N* = 99)	STUDY 2 Severely obese with T2DM (*N* = 146)
Age (years)		
Mean (SD)	48.2 (11.24)	56.4 (9.03)
Median, Min-Max	49.0, 20.0–68.0	59.0, 25.0–70.0
Sex, n (%)		
Male	28 (28.3)	60 (41.1)
Female	71 (71.7)	86 (58.9)
Race, n (%)		
White	79 (79.8)	101 (69.2)
Black or African American	13 (13.1)	42 (28.8)
Other or unknown	7 (7.1)	3 (2.0)
Ethnicity, n (%)		
Hispanic or Latino	20 (20.2)	37 (25.3)
Not Hispanic or Latino	79 (79.8)	109 (74.7)
BMI (kg/m2)		
Mean (SD)	40.9 (4.28)	40.3 (4.33)
Median, Min-Max	40.4, 34.1–50.6	40.0, 34.1–51.6

For each study, the psychometric analysis sample included all patients in the modified intent-to-treat clinical analysis data set who completed at least one DAILY EATS item at least 1 day at baseline and also at least 1 day in a follow-up week

*BMI* Body mass index, *SD* Standard deviation, *T2DM* Type 2 diabetes mellitus

Descriptive statistics of the supporting PRO measures used in the psychometric evaluation were reviewed (data not shown). The dominant baseline responses were “Moderate” on the PGIS-Hunger, the PGIS-Cravings, and the PGIS-Appetite; this supports patients acknowledge concerns in the key eating behavior concepts assessed on the DAILY EATS. Notably, most patients reported being “Completely satisfied” on the PGIS-Satiety in both studies, suggesting that patients were eating to being comfortably full. The baseline scores of the PRO measures addressing physical functioning and health-related quality of life tended to correspond to a better status in the sample without diabetes (Study 1) than the scores in the sample with T2DM (Study 2).

The trends in the responses of PGIS-Hunger, PGIS-Cravings, and PGIS-Appetite showed improvement from “Moderate” to “Mild” by EOT. In addition, by EOT patients on average showed some overall improvement on all the supporting measures in both studies.

### Descriptive statistics, missing data, and DAILY EATS structure

An examination of the item response distributions during the baseline weeks in Study 1 and Study 2 indicated little evidence of ceiling effects and no evidence of floor effects. Over the baseline week, the highest percentage of patients who reported a daily score of 10 on any day was from DAILY EATS Satiety (Item 5) in both studies (15.3% in Study 1; 19.9% in Study 2) (data not shown). The baseline weekly averages were indicative of moderate severities on eating-related concepts and ranged from 5.9 (Average Hunger [Item 1]) to 7.1 (Satiety [Item 5]) in Study 1 and from 5.0 (Average Hunger [Item 1]) to 6.9 (Satiety [Item 5]) in Study 2 (Table 8 in Appendix).

The average weekly change from baseline to EOT was an improvement (a decline for Items 1–4 and an increase for Item 5) of approximately − 1.1 points across the items in both studies; the change in Cravings (Item 3) was the largest at − 1.6 (Study 1), and the change in Satiety (Item 5) was the smallest at 0.3 and 0.0 points (Table 8 in Appendix).

Across evaluated time points and studies, more than 98% of patients completed all five items of the DAILY EATS for at least 6 days, indicating very good assessment compliance. No problematic completion differences were observed across the items. Missing simulation analyses in both studies showed that the 95% CIs of the SD of each item-level weekly score from partially complete data were still within the ±0.5 limits of the SD from complete data, despite the random loss of up to 6 daily responses. These results support the proposed missing rule for weekly scoring (requirement of at least 4 days of data per week).

Satiety (Item 5) scores performed differently than the other DAILY EATS items (i.e., low EFA loadings and weak inter-item correlation (Table 9 in Appendix) and Table 2) when evaluating the DAILY EATS structure. Further, because Average and Worst Hunger item scores were found to be potentially redundant (i.e., a high degree of collinearity due to overlapping content area), Worst Hunger (Item 2) was retained for further consideration instead of Average Hunger (Item 1). Subsequently, analyses of the DAILY EATS structure supported the computation of a three-item DAILY EATS composite, the EDI, as the average of the weekly scores for Worst Hunger (Item 2), Appetite (Item 3), and Cravings (Item 4) for both populations. It is recommended that Average Hunger (Item 1) and Satiety (Item 5) should be reported separately.

**Table 2:** Reliability and Structure of the Weekly DAILY EATS item and EDI Composite Scores

DAILY EATS	Cronbach’s Alpha	Test-retest reliability ICC (95% CI), n^a^		One-factor EFA loading
	Study 1/Study 2	Study 1	Study 2	Study 1/Study 2
Item 1. Average hunger	0.94/0.94	0.80 (0.62–0.90), 28	0.71 (0.54–0.82), 55	0.93*/0.95*
Item 2. Worst hunger	0.94/0.95	0.79 (0.60–0.90), 28	0.71 (0.53–0.83), 55	0.97*/0.95*
Item 3. Appetite	0.93/0.94	0.76 (0.55–0.88), 30	0.60 (0.39–0.74), 55	0.92*/0.91*
Item 4. Cravings	0.93/0.95	0.84 (0.66–0.93), 23	0.72 (0.56–0.83), 52	0.73*/0.76*
Item 5. Satiety	0.95/0.95	0.67 (0.36–0.85), 22	0.58 (0.41–0.72), 74	0.21*/0.22*
EDI	0.90/0.91	0.88 (0.76–0.94), 28	0.75 (0.54–0.86), 55	–

The EDI is computed as the mean of Worst Hunger (Item 2), Appetite (Item 3), and Cravings (Item 4) weekly average scores

*CI* Confidence interval, *EDI* Eating Drivers Index, *EFA* Exploratory factor analysis, *EOT* End of treatment, *ICC* Intraclass Correlation Coefficient, *PGIS* Patient Global Impression of Severity, *SE* Standard error, *T2DM* Type 2 diabetes mellitus

^a^The Study 1 data used were Week 15 (test) and Week 26/EOT (retest) for the subgroup of patients with no PGIS change; the Study 2 data used were baseline (test) and Week 12/EOT (retest) for the subgroup of patients with no PGIS change

* *P* <  0.05 for H0: loading = 0

### Reliability

Item-level test-retest reliability coefficients were above 0.7, except for Appetite (Item 3) in Study 2 and Satiety (Item 5) in both studies (Table 2). The smaller magnitude of the ICC for Satiety (Item 5) was expected since responses in both samples were high throughout the treatment period, reducing the scores’ variability across participants (hence the ICC) at each time point. Internal consistency reliability for the EDI was strong across studies and time points (all Cronbach’s coefficient alpha ≥0.80), providing evidence to support the relationships among the items to justify reporting a composite score. Test-retest reliability coefficients were greater than the 0.7 threshold for both studies for the EDI, indicating stability in the EDI scores.

### Validity

#### Construct validity

Correlations were computed between the weekly DAILY EATS items and EDI composite scores and supportive measures at EOT (Table 3). Correlation patterns observed were generally as hypothesized. Specifically, strong positive correlations were observed between the DAILY EATS items for Worst Hunger, Appetite, and Cravings and their corresponding PGIS items. Correlations between pairs of DAILY EATS items and PGIS referring to similar content were typically the largest observed. Also as expected, the correlations between the three DAILY EATS item scores and PGIS-PF were considerably lower than the correlations between the item scores and the eating-related PGIS items. EDI composite scores consistently showed moderate to strong correlations with PGIS-Hunger (0.30 ≤ r ≤ 0.68), PGIS-Cravings (0.33 ≤ r ≤ 0.77), and PGIS-Appetite (0.52 ≤ r ≤ 0.77) across time points and studies. These correlations were significant and much stronger than the correlations between the EDI composite and any other supporting measure (e.g., PGIS-Satiety, PGIS-PF). Small to moderate negative correlations were observed between the three DAILY EATS item and EDI composite scores and all the IWQOL-Lite scores. Trivial to small correlations were observed between the three DAILY EATS item and EDI composite scores and PROMIS PF SF scores. As expected, the correlations with BMI in Study 1 were trivial (near 0) and the correlations with BMI in Study 2 were small.

**Table 3:**
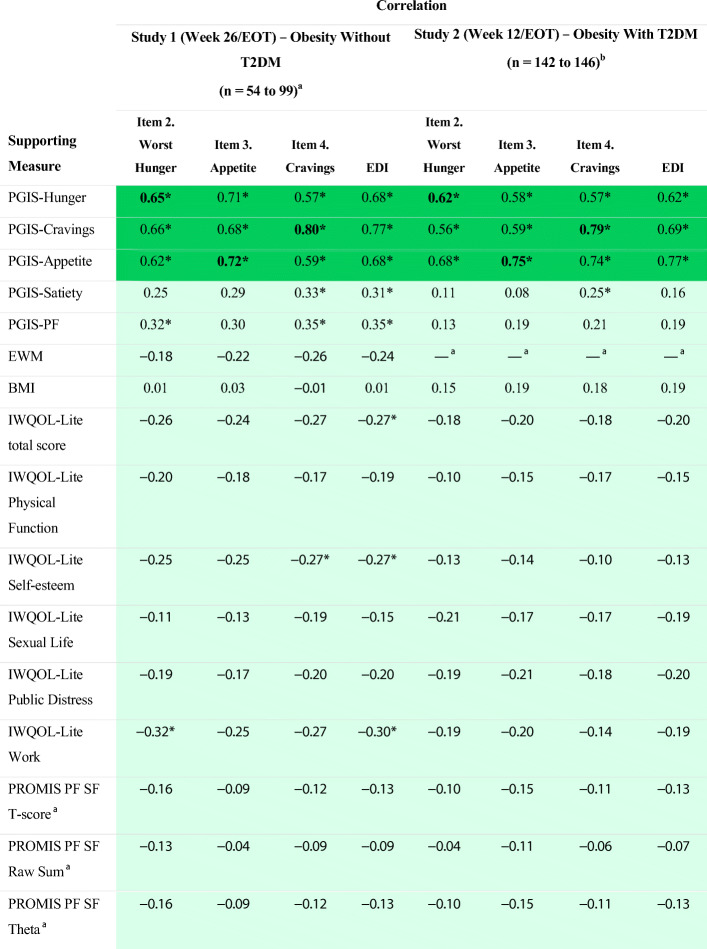
Construct Validity Correlations for DAILY EATS Scores

*BMI* body mass index, *EDI* Eating Drivers Index, *EOT* end of treatment, *EWM* ease of weight management, *IWQOL‑Lite* 31-item Impact of Weight on Quality of Life, *PGIS* Patient Global Impression of Status, *PGIS-PF* PGIS-Physical Functioning, *PROMIS PF SF* Patient-Reported Outcomes Measurement Information System Physical Function Short Form, *T2DM* type 2 diabetes mellitus

^a^Only Study 1 includes EWM, PROMIS PF SF 8b, and Week 26/EOT

^b^Study 2 uses PROMIS PF SF10a and Week 12/EOT

Bolded values indicate measures with corresponding concepts. Green highlight is indicative of patterns that support the construct validity hypotheses and blue highlight is indicative of patterns that were not supportive

*P < 0.01 for H0: *ρ* = 0

#### Known-groups validity

Known-groups ANOVAs were conducted to evaluate the discriminating ability of the three weekly DAILY EATS item and EDI composite scores at baseline and Week 26/EOT or Week 12/EOT. As hypothesized and shown in Table 4 for EOT, patients reporting “No …” or “Mild …” (e.g., hunger, appetite, cravings) on the corresponding PGIS (i.e., PGIS-Hunger, PGIS-Appetite, PGIS-Cravings) had lower (less severe) weekly DAILY EATS item and EDI composite scores on average than those with “Moderate …” or “Severe …” responses (*P* <  0.0001). As expected, the mean weekly DAILY EATS item and EDI composite scores increased (increased hunger, appetite, or cravings) as the PGIS level increased (increased hunger, appetite, or cravings), providing strong support for the discriminating ability of the individual DAILY EATS items and the EDI composite. Results were strongest for Cravings (Item 4) and the EDI composite, with the overall and all pairwise comparisons statistically significant at the *P* <  0.05 level.

**Table 4:** Known-Groups Results for the Weekly DAILY EATS item and the EDI Composite Scores

DAILY EATS Item/ Known-Group Results	Known-Groups ANOVA Results for DAILY EATS Scores at EOT ^a^							
	STUDY 1 – Obesity Without Diabetes				STUDY 2– Obesity With T2DM			
DAILY EATS Item 2. Worst Hunger								
PGIS-Hunger	n	LS Mean (SE)			n	LS Mean (SE)		
1 = No hunger	8	3.1 (0.56)			22	2.3 (0.36)		
2 = Mild hunger	42	4.7 (0.24)			80	4.2 (0.19)		
3 = Moderate hunger	35	6.0 (0.27)			38	5.6 (0.27)		
4 = Severe hunger	5	8.4 (0.71)			2	7.9 (1.18)		
ANOVA		LS Mean Difference (95% CI)	F/t	Adjusted *P* Value		LS Mean Difference (95% CI)	F/t	Adjusted P Value
Overall		–	16.10	< 0.0001		–	21.94	< 0.0001
1 vs. 2		−1.6 (−3.2 to 0.1)	−2.59	0.0651		− 2.0 (− 3.0 to −0.9)	−4.85	< 0.0001
1 vs. 3		−2.9 (− 4.6 to −1.2)	− 4.71	< 0.0001		−3.4 (−4.6 to −2.2)	−7.50	< 0.0001
1 vs. 4		−5.3 (− 7.7 to −2.9)	− 5.88	< 0.0001		— ^b^	— ^b^	— ^b^
2 vs. 3		−1.3 (− 2.3 to −0.4)	− 3.69	0.0023		−1.4 (−2.3 to − 0.5)	−4.27	0.0002
2 vs. 4		−3.7 (−5.7 to −1.7)	−4.97	< 0.0001		— ^b^	— ^b^	— ^b^
3 vs. 4		−2.4 (−4.4 to − 0.3)	−3.15	0.0135		— ^b^	— ^b^	— ^b^
DAILY EATS Item 3. Appetite								
PGIS-Appetite	n	LS Mean (SE)			n	LS Mean (SE)		
1 = No appetite	2	1.9 (1.10)			10	1.9 (0.44)		
2 = Small appetite	32	3.6 (0.27)			67	3.2 (0.17)		
3 = Moderate appetite	52	5.7 (0.22)			59	5.4 (0.18)		
4 = Very big appetite	4	8.5 (0.78)			6	7.0 (0.56)		
ANOVA		LS Mean Difference (95% CI)	F/t	Adjusted P Value		LS Mean Difference (95% CI)	F/t	Adjusted P Value
Overall		–	21.51	< 0.0001		–	42.43	< 0.0001
1 vs. 2		— ^b^	— ^b^	— ^b^		−1.3 (−2.6 to −0.1)	−2.82	0.0321
1 vs. 3		— ^b^	— ^b^	— ^b^		−3.5 (−4.7 to − 2.2)	−7.33	< 0.0001
1 vs. 4		— ^b^	— ^b^	— ^b^		−5.1 (−7.0 to − 3.2)	−7.13	< 0.0001
2 vs. 3		−2.1 (−3.0 to −1.1)	−5.98	< 0.0001		−2.1 (− 2.8 to − 1.5)	− 8.67	< 0.0001
2 vs. 4		— ^b^	— ^b^	— ^b^		−3.8 (−5.3 to −2.2)	−6.39	< 0.0001
3 vs. 4		—^b^	— ^b^	— ^b^		− 1.6 (−3.2 to − 0.0)	− 2.75	0.0404
DAILY EATS Item 4. Cravings								
PGIS-Cravings	n	LS Mean (SE)			n	LS Mean (SE)		
1 = No cravings	18	1.8 (0.39)			38	1.7 (0.24)		
2 = Mild cravings	38	3.8 (0.27)			65	3.8 (0.18)		
3 = Moderate cravings	26	6.0 (0.32)			32	5.4 (0.26)		
4 = Very strong cravings	8	8.0 (0.58)			7	8.3 (0.56)		
ANOVA		LS Mean Difference (95% CI)	F/t	Adjusted *P* Value		LS Mean Difference (95% CI)	F/t	Adjusted *P* Value
Overall		–	38.86	< 0.0001		–	58.39	< 0.0001
1 vs. 2		−2.0 (−3.3 to −0.8)	−4.35	0.0002		− 2.1 (− 2.9 to −1.3)	−6.87	< 0.0001
1 vs. 3		−4.2 (−5.6 to −2.9)	−8.47	< 0.0001		−3.7 (−4.6 to −2.7)	−10.33	< 0.0001
1 vs. 4		−6.3 (− 8.1 to −4.4)	−9.02	< 0.0001		−6.5 (−8.2 to −4.9)	− 10.73	< 0.0001
2 vs. 3		−2.2 (−3.3 to − 1.1)	−5.32	< 0.0001		−1.6 (−2.4 to −0.7)	−4.97	< 0.0001
2 vs. 4		−4.2 (−5.9 to −2.5)	−6.65	< 0.0001		−4.5 (− 6.0 to −2.9)	−7.57	< 0.0001
3 vs. 4		−2.0 (−3.8 to − 0.2)	− 3.05	0.0179		−2.9 (−4.5 to − 1.2)	−4.64	< 0.0001
EDI								
PGIS-Hunger	n	LS Mean (SE)			n	LS Mean (SE)		
1 = No hunger	8	2.4 (0.57)			22	2.3 (0.34)		
2 = Mild hunger	42	4.3 (0.25)			80	3.9 (0.18)		
3 = Moderate hunger	35	5.7 (0.27)			38	5.4 (0.26)		
4 = Severe hunger	5	8.1 (0.72)			2	8.0 (1.12)		
ANOVA		LS Mean Difference (95% CI)	F/t	Adjusted *P* Value		LS Mean Difference (95% CI)	F/t	Adjusted *P* Value
Overall		–	18.18	< 0.0001		–	22.44	< 0.0001
1 vs. 2		−1.9 (−3.6 to −0.3)	−3.13	0.0143		− 1.6 (−2.6 to − 0.6)	−4.19	0.0003
1 vs. 3		−3.4 (−5.0 to −1.7)	−5.34	< 0.0001		−3.1 (−4.2 to −2.0)	−7.31	< 0.0001
1 vs. 4		−5.7 (−8.1 to −3.2)	−6.24	< 0.0001		— ^b^	— ^b^	—^b^
2 vs. 3		−1.4 (−2.4 to −0.4)	−3.87	0.0013		−1.5 (−2.3 to − 0.7)	−4.82	< 0.0001
2 vs. 4		−3.8 (−5.8 to −1.7)	−4.96	< 0.0001		— ^b^	— ^b^	— ^b^
3 vs. 4		−2.3 (−4.4 to −0.3)	−3.06	0.0177		— ^b^	— ^b^	— ^b^

*ANOVA* Analysis of variance, *CI* Confidence interval, *EDI* Eating Drivers Index, *EOT* End of treatment, *LS* Least squares, *PGIS* Patient Global Impression of Severity, *PROMIS PF SF* Patient-Reported Outcomes Measurement Information System Physical Function Short Form, *SE* Standard error, *T2DM* Type 2 diabetes mellitus

^a^Study 1 EOT was Week 26; Study 2 EOT was Week 12

^b^*n* ≤ 5

### Responsiveness

The correlation coefficients for change from baseline to EOT scores between the three weekly DAILY EATS items and the EDI composite with a subset of the supporting measures are shown in Table 5. Table 10 in Appendix, provides the correlations for all supporting measures. Correlations between change in the weekly DAILY EATS item and EDI composite scores and change scores in the PGIS and PGIC measures, which assessed similar constructs, tended to be moderate to strong (|r| ≥ 0.30), as expected. Notably, Worst Hunger, Appetite, Cravings, and the EDI composite were more strongly correlated with change in eating-related PGIS and PGIC items than in other measures. Finally, correlations between the three DAILY EATS items and EDI composite and the IWQOL-Lite, PROMIS PF SF (8b and 10a), BMI, and weight change percentage tended to be small to moderate, as expected. Results for the additional longitudinal responsiveness evaluations are included within the (Table 11 in Appendix).

**Table 5:** DAILY EATS Ability to Detect Change Results

Change in supporting measure from BL to EOT ^a^	Correlation with change in DAILY EATS scores							
	STUDY 1 – obesity without T2DM (*n* = 76 to 91)				STUDY 2 – obesity with T2DM (*n* = 142			
	Item 2. Worst hunger	Item 3. Appetite	Item 4. Cravings	EDI	Item 2. Worst hunger	Item 3. Appetite	Item 4. Cravings	EDI
PGIS-Hunger	0.50*	0.59*	0.42*	0.54*	0.41*	0.46*	0.38*	0.45*
PGIS-Cravings	0.21	0.33*	0.45*	0.36*	0.44*	0.48*	0.48*	0.51*
PGIS-Appetite	0.59*	0.64*	0.59*	0.66*	0.55*	0.61*	0.58*	0.63*
PGIS-Satiety	0.28	0.35*	0.38*	0.37*	0.25*	0.30*	0.34*	0.32*
PGIS-PF	0.17	0.23	0.28	0.25	0.20	0.23*	0.17	0.22
PGIC-Hunger	0.49*	0.56*	0.59*	0.59*	0.43*	0.42*	0.46*	0.49*
PGIC-Cravings	0.48*	0.52*	0.58*	0.57*	0.39*	0.40*	0.43*	0.45*
EWM ^a^	−0.10	−0.16	−0.17	−0.15	— ^a^	— ^a^	— ^a^	— ^a^
BMI	0.35*	0.38*	0.35*	0.39*	0.29*	0.30*	0.31*	0.33*
% BMI	0.32*	0.36*	0.34*	0.37*	0.30*	0.32*	0.33*	0.35*
% Weight	0.32*	0.36*	0.34*	0.37*	0.30*	0.32*	0.33*	0.35*
IWQOL-Lite total score	−0.30*	−0.31*	−0.29*	−0.33*	− 0.29*	− 0.27*	− 0.27*	− 0.30*

Bolded values indicate measures with most-similar concepts (in the case of PGIS and PGIC measures)

*BL* Baseline, *EDI* Eating Drivers Index, *EOT* End of treatment, *EWM* Ease of weight management, *IWQOL-Lite* 31-item Impact of Weight on Quality of Life, *PGIC* Patient Global Impression of Change, *PGIS* Patient Global Impression of Severity, *PGIS-PF* PGIS-Physical Functioning, *PROMIS PF SF* Patient-Reported Outcomes Measurement Information System Physical Function Short Form, *T2DM* Type 2 diabetes mellitus

^a^Only Study 1 includes EWM, PROMIS PF SF 8b, and Week 26/EOT; Study 2 uses PROMIS PF SF 10a and Week 12/EOT

* *P* <  0.01 for H0: *ρ* = 0

### Interpretation of change

Anchor-based and distribution-based methods were used to estimate thresholds defining meaningful within-person change, or responder definitions, of the three DAILY EATS item and the EDI composite in individuals with severe obesity without diabetes (Study 1) and with T2DM (Study 2) after confirming the appropriateness of the candidate anchor measures. Table 6 displays the responder definition estimates characterizing improvement based on change in the corresponding PGIS and PGIC items, as well as the half–standard deviation and standard error of the measurement (SEM) estimates. Due to the small sample sizes in the 1-point deterioration PGIS subgroups and the “Moderately worse/Moderately hungrier/Moderately stronger cravings” PGIC subgroups, the estimation of thresholds identifying deterioration are not recommended using the current data. A larger sample is recommended to further investigate deterioration. Tables 12 and 13 in Appendix show the complete set of results.

**Table 6:** Range of Potential Meaningful Within-Person Change Thresholds Characterizing Improvement on DAILY EATS item and EDI Scores (Responder Definitions)

DAILY EATS/Anchor	Thresholds characterizing improvement	
	Study 1 Obesity without diabetes	Study 2 Obesity with T2DM
Item 2. Worst hunger		
PGIS-Hunger (1-point): mean (median), n	− 1.9 (− 1.6), 21	− 1.6 (− 1.5), 52
PGIC-Hunger (“Moderately”): mean (median), n	−1.0 (− 1.4), 17	− 1.1 (− 0.9), 29
Half-SD (SEM ^a^)	−0.87 (− 0.80)	− 0.93 (− 1.00)
Item 3. Appetite		
PGIS-Appetite (1-point): mean (median), n	−1.9 (− 1.8), 28	− 1.5 (− 1.3), 66
PGIC-Hunger ^b^ (“Moderately”): Mean (median), n	−1.0 (− 1.3), 17	− 1.1 (− 1.4), 29
Half-SD (SEM ^a^)	−0.82 (− 0.80)	− 0.88 (− 1.12)
Item 4. Cravings		
PGIS-Cravings (1-point): mean (median), n	−2.0 (−1.6), 26	−1.2 (− 1.1), 44
PGIC-Cravings (“Moderately”): mean (median), n	−0.9 (−1.3), 15	−1.2 (− 1.1), 39
Half-SD (SEM ^a^)	−0.97 (− 0.78)	−1.05 (− 1.11)
EDI		
PGIS-Hunger (1-point): Mean (median), n	−2.1 (− 2.1), 21	−1.5 (− 1.6), 52
PGIC-Hunger ^b^ (“Moderately”): Mean (median), n	−1.3 (− 1.6), 17	− 1.2 (− 1.3), 29
Half-SD (SEM ^a^)	−0.81 (− 0.56)	− 0.88 (− 0.88)

Study 1 uses change from baseline to Week 26/EOT; Study 2 uses change from baseline to Week 12/EOT

The sample sizes for deterioration (i.e., by 1-point in PGIS or moderately worsen in PGIC) were too small for reliable estimates of thresholds (generally, *n* ≤ 5)

*EDI* Eating Drivers Index, *EOT* End of treatment, *PGIC* Patient Global Impression of Change, *PGIS* Patient Global Impression of Status, *SD* Standard deviation at baseline, *SEM* Standard error of measurement, *T2DM* Type 2 diabetes mellitus

^a^Computed using SD at baseline and the intraclass correlation coefficients in 0

^b^PGIC-Hunger was selected as the anchor for this concept a priori

Bolded values indicate measures with corresponding concepts. Green highlight is indicative of patterns that support the construct validity hypotheses and blue highlight is indicative of patterns that were not supportive

*BMI* Body mass index, *EDI* Eating Drivers Index, *EOT* End of treatment, *EWM* Ease of weight management, *IWQOL-Lite* 31-item Impact of Weight on Quality of Life, *PGIS* Patient Global Impression of Status, *PGIS-PF* PGIS-Physical Functioning, *PROMIS PF SF* Patient-Reported Outcomes Measurement Information System Physical Function Short Form, *T2DM* Type 2 diabetes mellitus

^a^Only Study 1 includes EWM, PROMIS PF SF 8b, and Week 26/EOT

^b^Study 2 uses PROMIS PF SF 10a and Week 12/EOT

*P <  0.01 for H0: *ρ* = 0

The range of responder definitions, based on a 1-point improvement in the PGIS, the primary anchor, were higher than the range of estimates based on PGIC and the distribution-based methods. The thresholds estimated using anchor-based methods with Study 1 data tended to be slightly larger than the anchor-based thresholds estimated with Study 2 data. However, the SEM–based estimates were larger in Study 2 due to the lower ICCs (resulting from the test-retest evaluation timespan). Furthermore, all estimates were closer in magnitude across studies than within study using different methods (e.g., PGIS based, PGIC based, distribution based).

**Fig. 1:**
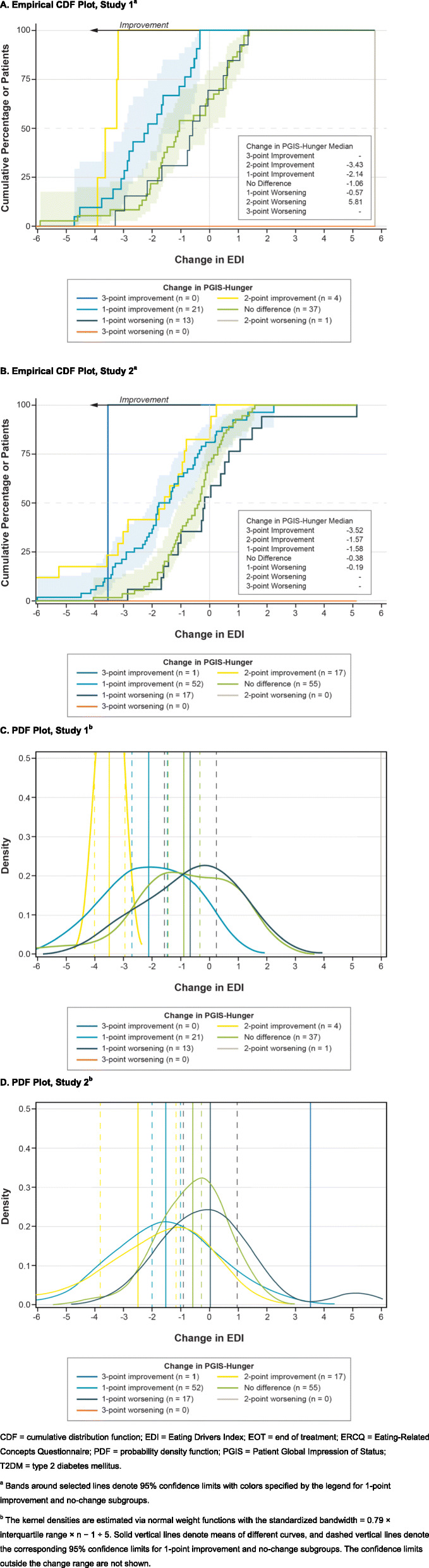
Changes From Baseline to EOT in EDI Composite by PGIS-Hunger. **a**. Empirical CDF Plot, Study 1^a^ . **b**. Empirical CDF Plot, Study 2^a^. **c**. PDF Plot, Study 1b. **d**. PDF Plot, Study 2^b^. CDF = cumulative distribution function; EDI = Eating Drivers Index; EOT = end of treatment; ERCQ = Eating-Related Concepts Questionnaire; PDF = probability density function; PGIS = Patient Global Impression of Status; T2DM = type 2 diabetes mellitus. ^a^ Bands around selected lines denote 95% confidence limits with colors specified by the legend for 1-point improvement and no-change subgroups. ^b^ The kernel densities are estimated via normal weight functions with the standardized bandwidth = 0.79 × interquartile range × n − 1 ÷ 5. Solid vertical lines denote means of different curves, and dashed vertical lines denote the corresponding 95% confidence limits for 1-point improvement and no-change subgroups. The confidence limits outside the change range are not shown

**Fig. 2:**
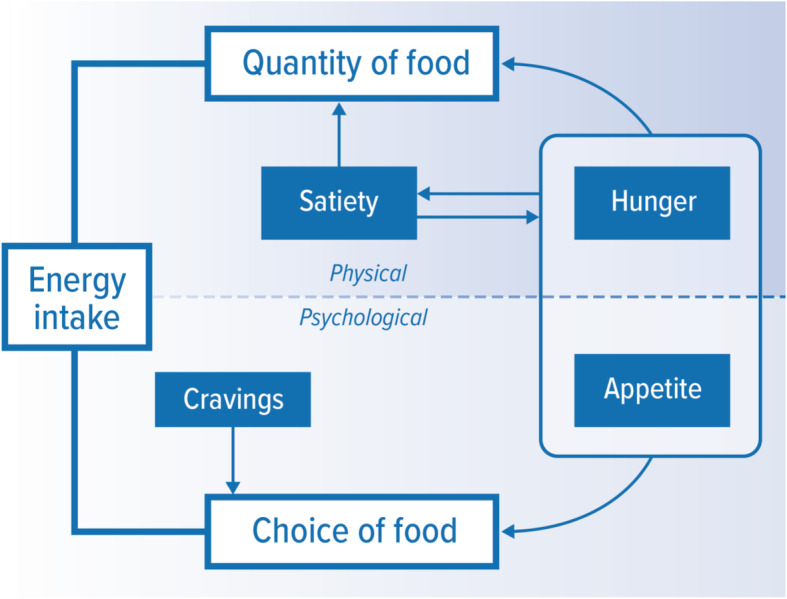
Conceptual Model

The CDF and PDF plots were reviewed to provide visual support of the primary anchor measures. For example, a greater proportion of patients with improvement in PGIS-Hunger also achieved improvement in the EDI composite from baseline to EOT across a range of possible response thresholds, as shown in the CDF curves for Studies 1 and 2 (Fig. 1a-b). The 1-point improvement (cyan blue) curve is clearly distinct from the no change (green) curves in each curve, providing support for the use of the 1-point improvement in PGIS as the primary anchor. In addition, PGIS-Hunger was adequately associated with the EDI composite change scores within each level of change in PGIS, as shown in the PDF plots for Studies 1 and 2 (Fig. 1c-d).

## Discussion

The purpose of this analysis was to evaluate the DAILY EATS measurement properties using data from two studies in severely obese adult patients with and without T2DM.

Descriptive statistics for the DAILY EATS item scores suggested adequate item performance with no limiting distributional anomalies or response biases in the daily and weekly average scores at baseline or EOT. Furthermore, the change in scores across time points was indicative of improvement during the study period in Average Hunger, Worst Hunger, Appetite, and Cravings items. In comparison, patients reported fairly high Satiety scores at baseline, suggesting they experienced a great deal of satisfaction with being “comfortably full” prior to treatment, and the scores over time provided evidence of maintenance of satisfaction.

A review of the structure of the DAILY EATS informed the preliminary scoring decisions. Average Hunger, Worst Hunger, Appetite, and Cravings items were strongly correlated, and the results suggest that these items can support the formation of a composite score. Scores for Average and Worst Hunger exhibited a high degree of collinearity, which may be viewed as redundant (overlapping content area); thus, the Worst Hunger item was retained in the composite instead of Average Hunger. The resulting composite, the EDI, is an average of the three item weekly scores. The item-level results also indicated that Satiety scores performed differently than the other DAILY EATS items (i.e., low loadings and weak inter-item correlation). These results corroborate the findings from the qualitative work with obese patients that the concept of satiety is distinct from the other eating-related factors. Given the importance of the concept of satiety, it is recommended that the DAILY EATS questionnaire retain the Satiety item and report it in addition to the other item and EDI composite scores. Due to the small sample sizes in the PGIS and PGIC subgroups for satiety, the estimation of thresholds identifying deterioration could not be evaluated using the current data. A larger sample is recommended to further investigate deterioration.

Overall, Average Hunger, Worst Hunger, Appetite, Cravings, and the EDI composite weekly and change scores demonstrated acceptable measurement properties. Internal consistency evidence was strong and supported the EDI composite. Test-retest reliability estimates were well above the recommended 0.70 threshold when using Study 1 data; ICCs based on Study 2 were not as strong, potentially owing to differences in the studies’ respective test-retest evaluation time points. Study 1 used a span of 9 weeks, and both time points were within the treatment period; Study 2 used a span of 14 weeks, in which the first time period occurred within the pretreatment phase and the second time period occurred within the treatment phase. The remaining properties focused on the EDI composite.

For construct validity, the patterns of correlations with other PRO measures were as hypothesized and consistent across the two studies, thus supporting the weekly DAILY EATS item scores and EDI composite scores and the constructs measured. Mean weekly item and composite scores also differed as anticipated and significantly across known groups based on the PGIS, providing evidence for the scores discriminating between meaningful groups. Lastly, the weekly item and composite scores demonstrated responsiveness based on the moderate to strong correlations of change observed with the related PGIS and PGIC measures, the moderate to large effect-size estimates of change, and the moderate to large magnitudes of change observed across levels of change in the PGIS and between PGIC improvement classification groups.

Finally, results of the anchor-based analyses using the PGIS provided evidence that changes ranging from − 1.5 (mean) to − 2.1 (mean or median) for the EDI composite were appropriate for identifying meaningful within-person improvement. Estimates based on the PGIC, a supportive anchor, tended to be lower in magnitude than the PGIS-based estimates for the items and EDI composite, and the distribution-based estimates were lower than the anchor-based values.

Along with the existing qualitative evidence supporting the measure’s content validity in these patient populations [4], the quantitative results provide further evidence that the DAILY EATS item and EDI composite scores are well-defined, reliable, sensitive, and valid for use in individuals with severe obesity with or without T2DM.

## Conclusions

The five-item DAILY EATS and its EDI composite exhibit content validity and good psychometric properties for assessing key factors related to eating. The DAILY EATS item and EDI composite scores shows similar performance among individuals with severe obesity alone and individuals with severe obesity and T2DM, providing a fit-for-purpose measure of eating-related behaviors. The proposed scoring algorithm and thresholds for meaningful change are recommended for both populations.

## Data Availability

Not applicable.

## Appendix

### Measures Relevant to the Psychometric Evaluation

**Table 7:** Measures Relevant to the Psychometric Evaluation

Outcome measure	Recall period	Time points used in evaluation
DAILY EATS Version 2.0	24 h	Study 1 Baseline (starting Week − 1 to the day before Day 1) Week 15 (Week 15 up to the day before Week 16) Week 26/EOT (Week 25 up to the day before Week 26) Study 2 Baseline (Week − 2 and the day before Day 1) Week 12/EOT (daily between the day after the Week 10 contact reminder and the day before Week 12)
PGIS-Hunger V2.0	7 days	Study 1 Week − 2, Week 15, and Week 26 Study 2 Week − 2 and Week 12
PGIS-Appetite V2.0	7 days	Study 1 Week − 2, Week 15, and Week 26 Study 2 Week − 2 and Week 12
PGIS-Cravings V2.0	7 days	Study 1 Week −2, Week 15, and Week 26 Study 2 Week − 2 and Week 12
PGIS-Satiety V2.0	7 days	Study 1 Week − 2, Week 15, and Week 26 Study 2 Week − 2 and Week 12
PGIS-Physical Functioning V2.0	7 days	Study 1 Week −2, Week 15, and Week 26 Study 2 Week − 2 and Week 12
PGIC-Hunger V2.0	7 days	Study 1 Week 15 and Week 26 Study 2 Week 12
PGIC-Cravings V2.0	7 days	Study 1 Week 15 and Week 26 Study 2 Week 12
PGIC-PF V2.0	7 days	Study 1 Week 15 and Week 26 Study 2 Week 12
IWQOL-Lite	Past week	Study 1 Week −2, Week 15, and Week 26 Study 2 Week − 2 and Week 12
EWM	Current	Study 1 Week −2, Week 15, and Week 26 Study 2 Not administered
PROMIS PF SF 8b	Does not have a reference period	Study 1 Week −2, Week 15, and Week 26 Study 2 Not administered
PROMIS PF SF 10a	Does not have a reference period	Study 1 Not administered Study 2 Week −2 and Week 12

*EOT* End of treatment, *EWM* Ease of weight management, *IWQOL-Lite* 31-item Impact of Weight on Quality of Life, *PF* Physical functioning, *PGIC* Patient Global Impression of Change, *PGIS* Patient Global Impression of Status, *PRO* Patient-reported outcome, *PROMIS* Patient-Reported Outcomes Measurement Information System, *SD* Standard deviation, *SF* Short form

### Descriptive Statistics for Supportive Measures

**Table 8:** DAILY EATS Weekly Scores Descriptive Statistics

Measure	n	Mean (SD)	Q1, Median, Q3	Min, Max	Floor/Ceiling %	Missing (%)
Study 1 (without T2DM)						
Baseline						
Item 1. Average hunger	99	5.9 (1.61)	4.7, 5.9, 7.0	1.4, 10.0	0.0/1.0	0 (0.0)
Item 2. Worst hunger	99	6.4 (1.75)	5.0, 6.6, 7.7	1.4, 10.0	0.0/1.0	0 (0.0)
Item 3. Appetite	99	6.2 (1.65)	5.0, 6.0, 7.3	1.4, 10.0	0.0/2.0	0 (0.0)
Item 4. Cravings	99	6.1 (1.95)	4.6, 5.9, 7.4	2.0, 10.0	0.0/2.0	0 (0.0)
Item 5. Satiety	99	7.1 (1.86)	6.0, 7.4, 8.4	2.0, 10.0	0.0/5.1	0 (0.0)
Week 26 (EOT)						
Item 1. Average hunger	91	4.8 (2.05)	3.3, 5.1, 6.3	0.0, 9.3	1.1/0.0	8 (8.1)
Item 2. Worst hunger	91	5.3 (1.93)	3.6, 5.3, 6.7	0.9, 9.9	0.0/0.0	8 (8.1)
Item 3. Appetite	91	5.0 (2.01)	3.4, 5.3, 6.6	0.3, 9.6	0.0/0.0	8 (8.1)
Item 4. Cravings	91	4.4 (2.46)	3.0, 4.6, 6.3	0.0, 9.7	2.2/0.0	8 (8.1)
Item 5. Satiety	91	7.5 (1.94)	6.1, 7.6, 9.2	3.0, 10.0	0.0/17.6	8 (8.1)
Change from baseline to week 26 (EOT)						
Item 1. Average hunger	91	−1.1 (2.12)	−2.4, −0.9, 0.3	−6.9, 5.4	—/—	8 (8.1)
Item 2. Worst hunger	91	−1.1 (1.99)	−2.6, − 1.0, 0.0	−6.0, 6.0	—/—	8 (8.1)
Item 3. Appetite	91	−1.1 (2.03)	−2.4, −0.9, 0.1	−5.3, 6.1	—/—	8 (8.1)
Item 4. Cravings	91	−1.6 (2.10)	−3.0, −1.6, −0.3	−6.7, 5.3	—/—	8 (8.1)
Item 5. Satiety	91	0.3 (2.04)	−0.7, 0.0, 1.3	−4.4, 8.0	—/—	8 (8.1)
Study 2 (with T2DM)						
Baseline						
Item 1. Average hunger	146	5.0 (1.77)	4.0, 5.0, 6.0	0.0, 8.6	2.1/0.0	0 (0.0)
Item 2. Worst hunger	146	5.4 (1.87)	4.3, 5.7, 6.9	0.0, 8.9	1.4/0.0	0 (0.0)
Item 3. Appetite	146	5.3 (1.76)	4.3, 5.3, 6.3	0.0, 8.7	0.7/0.0	0 (0.0)
Item 4. Cravings	146	4.9 (2.11)	3.6, 5.0, 6.4	0.0, 10.0	2.7/0.7	0 (0.0)
Item 5. Satiety	146	6.9 (2.06)	5.7, 7.0, 8.3	0.0, 10.0	0.7/9.6	0 (0.0)
Week 12 (EOT)						
Item 1. Average hunger	142	3.9 (1.82)	2.7, 4.1, 5.1	0.0, 8.9	4.2/0.0	4 (2.7)
Item 2. Worst hunger	142	4.4 (2.01)	3.1, 4.6, 5.9	0.0, 8.6	3.5/0.0	4 (2.7)
Item 3. Appetite	142	4.2 (1.89)	3.0, 4.3, 5.4	0.0, 9.1	2.8/0.0	4 (2.7)
Item 4. Cravings	142	3.8 (2.21)	2.4, 3.7, 5.3	0.0, 9.0	7.7/0.0	4 (2.7)
Item 5. Satiety	142	6.9 (2.35)	5.7, 7.1, 8.8	0.0, 10.0	2.1/10.6	4 (2.7)
Change from baseline to week 12 (EOT)						
Item 1. Average hunger	142	−1.0 (1.84)	−2.0, −0.7, 0.0	−7.6, 4.9	—/—	4 (2.7)
Item 2. Worst hunger	142	−1.1 (1.98)	−2.1, −0.8, 0.1	−7.9, 5.1	—/—	4 (2.7)
Item 3. Appetite	142	−1.1 (1.88)	−2.0, −1.0, 0.0	−8.2, 4.7	—/—	4 (2.7)
Item 4. Cravings	142	−1.1 (2.07)	−2.1, −1.1, 0.0	−9.1, 5.6	—/—	4 (2.7)
Item 5. Satiety	142	−0.0 (2.02)	−1.0, 0.0, 1.3	−6.1, 5.9	—/—	4 (2.7)

*EOT* end of treatment, *Q1* 25th percentile, *Q3* 75th percentile, *SD* standard deviation, *T2DM* type 2 diabetes mellitus

*Note*: For DAILY EATS weekly scores, floor is defined as score 0 and ceiling is defined as score 10

For each study, the psychometric analysis sample included all patients in the modified intent-to-treat clinical analysis data set who completed at least one DAILY EATS item at least 1 day at baseline and also at least 1 day in a follow-up week

### DAILY EATS Inter-Item Correlations

**Table 9:** Inter-item Correlations at Baseline and EOT, Study 1 and Study 2

Item	Inter-item Correlation				
	Item 1	Item 2	Item 3	Item 4	Item 5
Study 1, *n* = 91 to 99					
Item 1. Average hunger	–	0.93*	0.93*	0.82*	−0.23
Item 2. Worst hunger	0.91*	–	0.92*	0.77*	−0.12
Item 3. Appetite	0.83*	0.89*	–	0.80*	−0.17
Item 4. Cravings	0.63*	0.66*	0.73*	–	−0.28*
Item 5. Satiety	−0.05	0.06	0.10	−0.00	–
Study 2, n = 142 to 146					
Item 1. Average hunger	–	0.95*	0.90*	0.78*	0.11
Item 2. Worst Hunger	0.91*	–	0.90*	0.78*	0.16
Item 3. Appetite	0.86*	0.86*	–	0.81*	0.22*
Item 4. Cravings	0.73*	0.71*	0.75*	–	0.04
Item 5. Satiety	0.24*	0.34*	0.38*	0.21	–

*Note*: The baseline inter-item correlations are in the bottom left triangle below the main diagonal, and the end of treatment (Study 1: Week 26; Study 2: Week 12) inter-item correlations are in the top right triangle above the main diagonal

*EOT* end of treatment, *ERCQ* Eating-Related Concepts Questionnaire

* *P* <  0.01

### Responsiveness

**Table 10:** DAILY EATS Worst Hunger, Appetite, Cravings, and EDI Ability to Detect Change Results: Correlational Analysis of Change

Supporting measure	Correlation with change in DAILY EATS score							
	STUDY 1 – obesity without T2DM (*n* = 76 to 91)				STUDY 2 – obesity with T2DM (*n* = 142			
	Item 2. Worst hunger	Item 3. Appetite	Item 4. Cravings	EDI	Item 2. Worst hunger	Item 3. Appetite	Item 4. Cravings	EDI
Change from baseline to EOT a								
PGIS-Hunger	0.50*	0.59*	0.42*	0.54*	0.41*	0.46*	0.38*	0.45*
PGIS-Cravings	0.21	0.33*	0.45*	0.36*	0.44*	0.48*	0.48*	0.51*
PGIS-Appetite	0.59*	0.64*	0.59*	0.66*	0.55*	0.61*	0.58*	0.63*
PGIS-Satiety	0.28	0.35*	0.38*	0.37*	0.25*	0.30*	0.34*	0.32*
PGIS-PF	0.17	0.23	0.28	0.25	0.20	0.23*	0.17	0.22
PGIC-Hunger	0.49*	0.56*	0.59*	0.59*	0.43*	0.42*	0.46*	0.49*
PGIC-Cravings	0.48*	0.52*	0.58*	0.57*	0.39*	0.40*	0.43*	0.45*
PGIC-PF	0.26	0.17	0.20	0.22	0.17	0.17	0.20	0.20
EWM a	−0.10	−0.16	−0.17	−0.15	— a	— a	— a	— a
BMI	0.35*	0.38*	0.35*	0.39*	0.29*	0.30*	0.31*	0.33*
% BMI	0.32*	0.36*	0.34*	0.37*	0.30*	0.32*	0.33*	0.35*
% Weight	0.32*	0.36*	0.34*	0.37*	0.30*	0.32*	0.33*	0.35*
IWQOL-Lite total score	−0.30*	−0.31*	− 0.29*	− 0.33*	− 0.29*	− 0.27*	− 0.27*	−0.30*
IWQOL-Lite Physical Function	−0.27*	− 0.27	− 0.27*	−0.29*	−0.17	−0.15	−0.17	−0.18
IWQOL-Lite self-esteem	−0.18	−0.22	−0.18	−0.21	− 0.25*	−0.28*	−0.24*	−0.28*
IWQOL-Lite sexual life	−0.22	−0.21	− 0.20	− 0.23	− 0.30*	−0.24*	−0.29*	−0.31*
IWQOL-Lite public distress	−0.16	−0.20	−0.24	−0.22	−0.19	−0.20	−0.18	−0.21
IWQOL-Lite work	−0.32*	−0.30*	−0.22	−0.30*	−0.28*	−0.23*	−0.19	− 0.25*
PROMIS PF SF T-score a	−0.23	−0.19	−0.23	−0.24	−0.24*	−0.26*	−0.17	−0.24*
PROMIS PF SF raw sum a	−0.23	−0.15	−0.20	− 0.21	−0.34*	−0.36*	−0.25*	−0.34*
PROMIS PF SF Theta a	−0.23	−0.19	−0.23	−0.24	−0.24*	−0.26*	−0.17	−0.24*

### Longitudinal Responsiveness

Patterns of mean change in the weekly DAILY EATS item scores across the levels of the change in the corresponding PGIS and PGIC provide supportive evidence for the responsiveness of the EDI composite score. As an item-level example, the largest average weekly Worst Hunger (Item 2) score change reflecting improvement (negative change) was in the improved PGIS-Hunger subgroup (least square mean, − 2.1 and − 1.8) and this value was significantly larger than the mean change in the stable subgroup (least square mean, − 0.7 and − 0.4; *P* = 0.0072). In addition to the mean differences, the effect-size estimates of change for the EDI composite based on the SD at baseline, the SD of change, and between the PGIS subgroups are moderate to large, at least 0.50 or greater.

**Table 11:** DAILY EATS Ability to Detect Change: Longitudinal Construct Validity

DAILY EATS/Subgroup	ANOVA Results Predicting Change in DAILY EATS Change Scores (Baseline to EOT ^a^) from Subgroup							
	Study 1 – Obesity Without T2DM				Study 2 – Obesity With T2DM			
DAILY EATS Item 2. Worst Hunger								
PGIS-Hunger	n	LS Mean (SE)			n	LS Mean (SE)		
Improved	25	−2.1 (0.35)			70	−1.8 (0.20)		
Stable	37	−0.7 (0.29)			55	−0.4 (0.23)		
Worsened	14	−0.2 (0.47)			17	0.2 (0.41)		
Comparisons		Cohen’s d	F/t	Adjusted *P* Value		Cohen’s d	F/t	Adjusted P Value
Overall		–	7.18	0.0014		–	15.76	< 0.0001
Improved vs. Stable		−0.8	− 3.15	0.0072		−0.8	−4.66	< 0.0001
Improved vs. Worsened		−1.1	−3.33	0.0041		−1.1	−4.36	< 0.0001
Stable vs. Worsened		−0.3	−0.94	0.7228		−0.3	−1.22	0.5344
PGIC-Hunger	n	LS Mean (SE)			n	LS Mean (SE)		
Improved	67	−1.5 (0.23)			106	−1.4 (0.17)		
Stable	12	−0.2 (0.55)			24	−0.0 (0.35)		
Worsened	11	0.0 (0.57)			12	0.4 (0.50)		
Comparisons		Cohen’s d	F/t	Adjusted P Value		Cohen’s d	F/t	Adjusted P Value
Overall		–	5.16	0.0076		–	10.41	< 0.0001
Improved vs. Stable		−0.8	−2.32	0.0665		−0.8	−3.54	0.0016
Improved vs. Worsened		−0.9	−2.54	0.0383		−1.0	−3.34	0.0032
Stable vs. Worsened		−0.1	− 0.24	0.9936		−0.2	−0.61	0.9033
ESE (SD of Baseline Score)		−0.7 (1.75)				−0.6 (1.77)		
SRM (SD of Change Score)		−0.6 (1.99)				−0.5 (1.84)		
DAILY EATS Item 3. Appetite								
PGIS-Appetite	n	LS Mean (SE)			n	LS Mean (SE)		
Improved	33	−2.1 (0.29)			83	−1.8 (0.18)		
Stable	38	−0.5 (0.27)			55	−0.1 (0.23)		
Worsened	5	1.8 (0.74)			4	0.3 (0.84)		
Comparisons		Cohen’s d	F/t	Adjusted P Value		Cohen’s d	F/t	Adjusted P Value
Overall		–	16.29	< 0.0001		–	18.50	< 0.0001
Improved vs. Stable		−1.0	−4.12	0.0003		−1.0	−5.85	< 0.0001
Improved vs. Worsened		−2.4	−4.91	< 0.0001		−1.2	−2.43	0.0478
Stable vs. Worsened		−1.4	−2.89	0.0151		−0.2	−0.44	0.9605
PGIC-Hunger	n	LS Mean (SE)			n	LS Mean (SE)		
Improved	67	−1.6 (0.23)			106	−1.4 (0.18)		
Stable	12	−0.0 (0.54)			24	−0.1 (0.37)		
Worsened	11	0.3 (0.57)			12	−0.6 (0.53)		
Comparisons		Cohen’s d	F/t	Adjusted *P* Value		Cohen’s d	F/t	Adjusted *P* Value
Overall		–	7.32	0.0012		–	5.80	0.0038
Improved vs. Stable		−0.9	−2.65	0.0286		−0.8	−3.24	0.0044
Improved vs. Worsened		−1.2	−3.12	0.0074		−0.5	−1.47	0.3709
Stable vs. Worsened		−0.2	− 0.44	0.9605		0.3	0.81	0.8069
ESE (SD of Baseline Score)		−0.7 (1.65)				−0.6 (1.76)		
SRM (SD of Change Score)		−0.5 (2.03)				−0.6 (1.88)		
DAILY EATS Item 4. Cravings								
PGIS-Cravings	n	LS Mean (SE)			n	LS Mean (SE)		
Improved	40	−2.4 (0.32)			76	−1.9 (0.22)		
Stable	31	−0.8 (0.36)			52	−0.3 (0.27)		
Worsened	5	−0.4 (0.90)			14	0.1 (0.51)		
Comparisons		Cohen’s d	F/t	Adjusted *P* Value		Cohen’s d	F/t	Adjusted *P* Value
Overall		–	6.13	0.0035		–	13.01	< 0.0001
Improved vs. Stable		−0.8	−3.21	0.0059		−0.7	−4.46	< 0.0001
Improved vs. Worsened		−1.0	−2.07	0.1204		−0.9	−3.52	0.0018
Stable vs. Worsened		−0.2	− 0.44	0.9599		−0.2	−0.73	0.8470
PGIC-Cravings	n	LS Mean (SE)			n	LS Mean (SE)		
Improved	58	−2.2 (0.25)			104	−1.5 (0.19)		
Stable	17	−0.9 (0.46)			24	−0.2 (0.40)		
Worsened	15	−0.1 (0.49)			14	0.2 (0.53)		
Comparisons		Cohen’s d	F/t	Adjusted *P* Value		Cohen’s d	F/t	Adjusted *P* Value
Overall		–	9.13	0.0003		–	7.58	0.0007
Improved vs. Stable		−0.7	−2.57	0.0349		−0.6	−2.81	0.0170
Improved vs. Worsened		−1.1	−3.89	0.0006		−0.8	−3.09	0.0073
Stable vs. Worsened		−0.4	−1.17	0.5669		−0.2	−0.72	0.8527
ESE (SD of Baseline Score)		−0.8 (1.95)				−0.5 (2.11)		
SRM (SD of Change Score)		−0.7 (2.10)				−0.5 (2.07)		
EDI								
PGIS-Hunger	n	LS Mean (SE)			n	LS Mean (SE)		
Improved	25	−2.3 (0.34)			70	−1.8 (0.20)		
Stable	37	−0.9 (0.28)			55	−0.6 (0.23)		
Worsened	14	−0.2 (0.45)			17	0.0 (0.41)		
Comparisons		Cohen’s d	F/t	Adjusted *P* Value		Cohen’s d	F/t	Adjusted *P* Value
Overall		–	8.56	0.0005		–	11.98	< 0.0001
Improved vs. Stable		−0.9	−3.29	0.0047		−0.7	−3.91	0.0004
Improved vs. Worsened		−1.3	−3.75	0.0011		−1.0	− 3.97	0.0004
Stable vs. Worsened		−0.4	−1.28	0.4995		−0.4	−1.33	0.4625
PGIC-Hunger	n	LS Mean (SE)			n	LS Mean (SE)		
Improved	67	−1.7 (0.21)			106	−1.4 (0.17)		
Stable	12	−0.2 (0.50)			24	−0.0 (0.35)		
Worsened	11	0.0 (0.52)			12	−0.3 (0.50)		
Comparisons		Cohen’s d	F/t	Adjusted P Value		Cohen’s d	F/t	Adjusted P Value
Overall		–	8.19	0.0006		–	7.91	0.0006
Improved vs. Stable		−1.0	−2.94	0.0125		−0.8	−3.60	0.0013
Improved vs. Worsened		−1.1	−3.18	0.0061		−0.7	−2.17	0.0922
Stable vs. Worsened		−0.1	− 0.27	0.9906		0.2	0.43	0.9628
ESE (SD of Baseline Score)		−0.8 (1.63)				− 0.6 (1.76)		
SRM (SD of Change Score)		−0.7 (1.88)				−0.6 (1.82)		

For each study, the psychometric analysis sample included all patients in the modified intent-to-treat clinical analysis data set who completed at least one DAILY EATS item at least 1 day at baseline and also at least 1 day in a follow-up week

*ANOVA* Analysis of variance, *EDI* Eating Drivers Index, *EOT* End of treatment, *ESE* Effect-size estimate, *F* F-statistic, *PF* Physical functioning, *PGIC* Patient Global Impression of Change, *PGIS* Patient Global Impression of Status, *PROMIS* Patient-Reported Outcomes Measurement Information System, *SD* Standard deviation, *SE* Standard error, *SF* Short form, *SRM* Standardized response mean, *t* T-statistic, *T2DM* Type 2 diabetes mellitus

^a^Only study Study 1 includes ease of weight management, PROMIS PF SF 8b, and Week 26/EOT; Study 2 uses PROMIS PF SF 10a and Week 12/EOT

### Interpretation of Change: Changes From Baseline to End of Treatment in DAILY EATS Items and Anchor Measures

**Table 12:** Interpretation of Change: Study 1, Severely Obese Without T2DM

Score/Methods	Changes in DAILY EATS from BL to EOT			
EDI				
Anchor-based Methods	n	Mean (SD)	Q1, Median, Q3	Min-Max
Based on PGIS-Hunger				
3-point improvement	0	–	–	–
2-point improvement	4	−3.5 (0.34)	− 3.8, − 3.4, − 3.2	− 3.9 - -3.2
1-point improvement	21	−2.1 (1.36)	−3.0, − 2.1, − 0.9	−4.7 - -0.3
No difference	37	−0.9 (1.66)	− 1.8, − 1.1, 0.6	−5.9 - 1.4
1-point worsening	13	−0.7 (1.47)	−1.7, − 0.6, 0.5	−3.3 - 1.4
2-point worsening	1	5.8 (−)	5.8, 5.8, 5.8	5.8–5.8
3-point worsening	0	–	–	–
Based on PGIC-Hunger				
1 = Much less hungry	27	−2.9 (1.41)	−3.9, −3.0, − 2.0	−5.9 - 0.0
2 = Moderately less hungry	17	−1.3 (1.44)	−2.3, − 1.6, −0.4	− 3.8 - 1.1
3 = A little less hungry	23	− 0.7 (1.31)	−1.7, − 0.6, 0.5	−4.6 - 1.4
4 = No change	12	− 0.2 (1.04)	−0.8, − 0.4, 0.6	−1.8 - 1.4
5 = A little hungrier	5	0.2 (3.54)	− 2.1, − 0.6, 1.0	− 3.3 - 5.8
6 = Moderately hungrier	5	0.3 (1.00)	− 0.0, 0.6, 1.2	− 1.2 - 1.2
7 = Much hungrier	1	− 2.0 (−)	− 2.0, − 2.0, − 2.0	−2.0 - -2.0
Distribution-based Methods		Estimate		
Half-SD		0.81		
SEM		0.56		
DAILY EATS Item 2. Worst Hunger				
Anchor-based Methods	n	Mean (SD)	Q1, Median, Q3	Min-Max
Based on PGIS-Hunger				
3-point improvement	0	–	–	–
2-point improvement	4	−3.2 (0.62)	−3.6, − 3.4, − 2.8	− 3.7 - -2.3
1-point improvement	21	−1.9 (1.43)	−2.9, − 1.6, − 0.7	−5.3 - 0.1
No difference	37	−0.7 (1.77)	− 1.4, − 0.7, 0.9	−6.0 - 2.0
1-point worsening	13	− 0.7 (1.34)	− 1.1, − 0.7, 0.0	− 2.7 - 1.3
2-point worsening	1	6.0 (−)	6.0, 6.0, 6.0	6.0–6.0
3-point worsening	0	–	–	–
Based on PGIC-Hunger				
1 = Much less hungry	27	−2.6 (1.70)	−3.9, − 2.6, − 1.3	−6.0 - 0.1
2 = Moderately less hungry	17	− 1.0 (1.76)	−2.4, − 1.4, − 0.3	−3.6 - 2.0
3 = A little less hungry	23	−0.6 (1.63)	−1.6, − 0.7, 0.6	−4.9 - 3.1
4 = No change	12	− 0.2 (0.93)	− 0.8, − 0.5, 0.6	−1.4 - 1.4
5 = A little hungrier	5	0.1 (3.64)	− 2.7, − 1.0, 1.1	− 2.7 - 6.0
6 = Moderately hungrier	5	0.5 (1.00)	0.0, 0.9, 1.0	− 1.0 - 1.6
7 = Much hungrier	1	−2.9 (−)	− 2.9, − 2.9, − 2.9	− 2.9 - -2.9
Distribution-based Methods		Estimate		
Half-SD		0.87		
SEM		0.80		
DAILY EATS Item 3. Appetite				
Anchor-based Methods	n	Mean (SD)	Q1, Median, Q3	Min-Max
Based on PGIS-Appetite				
3-point improvement	0	–	–	–
2-point improvement	5	−3.4 (1.07)	−4.3, − 3.0, −2.9	−4.7 - -2.1
1-point improvement	28	− 1.9 (1.86)	−3.3, − 1.8, − 0.8	−5.0 - 2.0
No difference	38	−0.5 (1.28)	− 1.1, − 0.3, 0.4	− 3.4 - 2.1
1-point worsening	5	1.8 (2.79)	0.6, 1.4, 2.3	− 1.4 - 6.1
2-point worsening	0	–	–	–
3-point worsening	0	–	–	–
Based on PGIC-Hunger				
1 = Much less hungry	27	−2.8 (1.62)	−4.3, − 2.9, − 2.0	−5.1 - 0.1
2 = Moderately less hungry	17	−1.0 (1.66)	−2.3, − 1.3, −0.3	− 3.4 - 2.0
3 = A little less hungry	23	− 0.6 (1.42)	−1.1, − 0.7, 0.4	− 5.3 - 1.9
4 = No change	12	−0.0 (1.55)	−1.2, 0.1, 1.3	−2.4 - 2.3
5 = A little hungrier	5	0.4 (3.55)	−1.4, −0.1, 0.9	− 3.3 - 6.1
6 = Moderately hungrier	5	0.5 (1.13)	0.3, 0.4, 0.9	−1.0 - 2.1
7 = Much hungrier	1	−1.3 (−)	− 1.3, − 1.3, − 1.3	− 1.3 - -1.3
Distribution-based Methods		Estimate		
Half-SD		0.82		
SEM		0.80		
DAILY EATS Item 4. Cravings				
Anchor-based Methods	n	Mean (SD)	Q1, Median, Q3	Min-Max
Based on PGIS-Cravings				
3-point improvement	3	−3.8 (1.57)	−5.3, − 3.9, − 2.1	− 5.3 - -2.1
2-point improvement	11	−3.0 (1.37)	−4.1, − 2.9, − 1.6	− 5.1 - -1.1
1-point improvement	26	−2.0 (1.77)	− 3.4, − 1.6, − 0.9	− 5.3 - 1.0
No difference	31	−0.8 (2.29)	− 2.3, − 0.7, 0.6	−6.7 - 5.3
1-point worsening	5	− 0.4 (2.35)	− 0.4, 0.3, 0.4	−4.3 - 2.0
2-point worsening	0	–	–	–
3-point worsening	0	–	–	–
Based on PGIC-Cravings				
1 = Much less hungry	28	−3.5 (1.58)	−4.6, − 3.5, −2.9	−6.7 - 0.4
2 = Moderately less hungry	15	−0.9 (1.53)	−2.1, − 1.3, 0.7	−3.0 - 2.0
3 = A little less hungry	15	− 1.2 (1.17)	− 1.9, − 1.4, − 0.3	− 3.6 - 1.0
4 = No change	17	− 0.9 (1.64)	− 1.7, − 1.3, − 0.3	−3.1 - 4.0
5 = A little hungrier	9	0.3 (2.24)	−0.6, 0.1, 0.9	−2.4 - 5.3
6 = Moderately hungrier	3	0.4 (0.72)	−0.3, 0.3, 1.1	−0.3 - 1.1
7 = Much hungrier	3	−1.8 (2.15)	−3.9, −2.0, 0.4	− 3.9 - 0.4
Distribution-based Methods		Estimate		
Half-SD		0.97		
SEM		0.78		

**Table 13:** Interpretation of Change: Study 2, Severely Obese With T2DM

Score/Methods	Changes in DAILY EATS from BL to EOT			
EDI				
Anchor-based Methods	n	Mean (SD)	Q1, Median, Q3	Min-Max
Based on PGIS-Hunger				
3-point improvement	1	−3.5 (−)	− 3.5, − 3.5, − 3.5	− 3.5 - -3.5
2-point improvement	17	−2.5 (2.58)	−3.2, − 1.6, − 0.9	−8.3 - 0.2
1-point improvement	52	−1.5 (1.73)	− 2.8, − 1.6, − 0.4	−6.0 - 2.2
No difference	55	− 0.6 (1.14)	− 1.3, − 0.4, 0.3	−4.0 - 1.6
1-point worsening	17	0.0 (1.80)	−1.1, − 0.2, 0.7	−2.9 - 5.1
2-point worsening	0	–	–	–
3-point worsening	0	–	–	–
Based on PGIC-Hunger				
1 = Much less hungry	50	−2.0 (2.06)	−3.3, −1.8, −0.4	−8.3 - 1.5
2 = Moderately less hungry	29	−1.2 (1.24)	− 1.9, − 1.3, − 0.1	−4.0 - 1.0
3 = A little less hungry	27	−0.6 (1.21)	−1.3, − 0.5, 0.2	−3.0 - 1.6
4 = No change	24	− 0.0 (1.35)	− 0.5, 0.2, 0.6	− 4.5 - 2.2
5 = A little hungrier	1	1.8 (−)	1.8, 1.8, 1.8	1.8–1.8
6 = Moderately hungrier	8	− 0.9 (1.09)	− 1.5, − 1.2, − 0.6	− 2.0 - 1.5
7 = Much hungrier	3	0.6 (3.97)	−2.2, − 1.1, 5.1	− 2.2 - 5.1
Distribution-based Methods		Estimate		
Half-SD		0.88		
SEM		0.88		
DAILY EATS Item 2. Worst Hunger				
Anchor-based Methods	n	Mean (SD)	Q1, Median, Q3	Min-Max
Based on PGIS-Hunger				
3-point improvement	1	−3.1 (−)	− 3.1, − 3.1, − 3.1	− 3.1 - -3.1
2-point improvement	17	−2.2 (2.61)	−3.2, − 1.1, − 0.4	−7.9 - 0.7
1-point improvement	52	− 1.6 (2.00)	− 3.0, − 1.5, − 0.2	−6.6 - 2.7
No difference	55	− 0.5 (1.37)	− 1.4, − 0.6, 0.4	−3.9 - 2.6
1-point worsening	17	0.2 (1.82)	−0.7, − 0.1, 1.1	−2.7 - 5.1
2-point worsening	0	–	–	–
3-point worsening	0	–	–	–
Based on PGIC-Hunger				
1 = Much less hungry	50	−2.0 (2.20)	−3.1, −1.9, −0.3	−7.9 - 2.6
2 = Moderately less hungry	29	−1.1 (1.30)	− 2.1, − 0.9, − 0.1	−3.9 - 0.7
3 = A little less hungry	27	−0.6 (1.61)	− 1.7, − 0.7, 0.6	− 3.6 - 2.6
4 = No change	24	−0.1 (1.74)	− 0.9, 0.1, 1.0	− 6.1 - 2.7
5 = A little hungrier	1	2.1 (−)	2.1, 2.1, 2.1	2.1–2.1
6 = Moderately hungrier	8	− 0.6 (0.88)	− 1.0, − 0.7, − 0.6	−1.6 - 1.4
7 = Much hungrier	3	0.4 (4.21)	−2.9, −1.1, 5.1	− 2.9 - 5.1
Distribution-based Methods		Estimate		
Half-SD		0.93		
SEM		1.00		
DAILY EATS Item 3. Appetite				
Anchor-based Methods	n	Mean (SD)	Q1, Median, Q3	Min-Max
Based on PGIS-Appetite				
3-point improvement	1	−8.2 (−)	−8.2, −8.2, − 8.2	−8.2 - -8.2
2-point improvement	16	−3.0 (1.94)	−3.7, −2.9, − 1.6	− 8.0 - -0.2
1-point improvement	66	−1.5 (1.47)	−2.1, − 1.3, − 0.4	−6.0 - 1.6
No difference	55	− 0.1 (1.48)	− 1.1, − 0.1, 0.6	− 3.9 - 4.7
1-point worsening	4	0.3 (1.45)	−1.0, 0.4, 1.5	− 1.3 - 1.6
2-point worsening	0	–	–	–
3-point worsening	0	–	–	–
Based on PGIC-Hunger				
1 = Much less hungry	50	−2.0 (2.07)	−3.0, −1.4, −0.6	−8.2 - 1.9
2 = Moderately less hungry	29	−1.1 (1.35)	− 2.1, − 1.4, 0.0	− 3.9 - 1.1
3 = A little less hungry	27	− 0.5 (1.27)	−1.4, − 0.6, 0.4	−3.1 - 1.6
4 = No change	24	−0.1 (1.47)	− 1.1, 0.0, 0.7	−4.3 - 3.3
5 = A little hungrier	1	2.4 (−)	2.4, 2.4, 2.4	2.4–2.4
6 = Moderately hungrier	8	− 1.1 (1.32)	− 1.9, − 1.4, − 0.8	− 2.4 - 1.9
7 = Much hungrier	3	−0.2 (4.52)	− 4.1, − 1.3, 4.7	−4.1 - 4.7
Distribution-based Methods		Estimate		
Half-SD		0.88		
SEM		1.12		
DAILY EATS Item 4. Cravings				
Anchor-based Methods	n	Mean (SD)	Q1, Median, Q3	Min-Max
Based on PGIS-Cravings				
3-point improvement	7	−3.0 (4.12)	−8.0, − 1.7, 0.7	−9.1 - 1.6
2-point improvement	25	−2.8 (1.89)	−4.1, −3.1, − 1.7	−5.7 - 1.3
1-point improvement	44	−1.2 (1.40)	− 1.9, − 1.1, − 0.3	− 4.0 - 1.6
No difference	52	−0.3 (1.63)	− 1.6, − 0.4, 0.8	−3.0 - 4.7
1-point worsening	13	0.2 (1.98)	−0.9, − 0.4, 1.1	− 2.0 - 5.6
2-point worsening	1	−1.7 (−)	− 1.7, − 1.7, − 1.7	− 1.7 - -1.7
3-point worsening	0	–	–	–
Based on PGIC-Cravings				
1 = Much less hungry	44	− 2.1 (2.30)	−3.4, − 1.6, − 0.2	− 9.1 - 1.6
2 = Moderately less hungry	39	−1.2 (1.68)	− 2.4, − 1.1, − 0.1	− 4.6 - 2.4
3 = A little less hungry	21	−0.8 (1.41)	−1.9, − 1.3, − 0.1	− 2.7 - 2.3
4 = No change	24	− 0.2 (1.30)	− 1.2, − 0.2, 0.9	− 3.0 - 1.6
5 = A little hungrier	7	0.8 (2.36)	− 1.0, 0.6, 3.0	− 1.9 - 4.7
6 = Moderately hungrier	4	− 1.6 (2.50)	− 3.3, − 1.3, 0.1	−4.9 - 1.1
7 = Much hungrier	3	1.3 (3.88)	− 2.0, 0.3, 5.6	− 2.0 - 5.6
Distribution-based Methods		Estimate		
Half-SD		1.05		
SEM		1.11		

## Abbreviations

ANOVA: Analysis of variance
BMI: Body mass index
CDF: Cumulative distribution function
EDI: Eating Drivers Index
EOT: End of treatment
EWM: Ease of Weight Management
ICC: Intraclass correlation coefficient
IWQOL-Lite: 31-item Impact of Weight on Quality of Life-Lite
PDF: Probability density function
PGIC: Patient Global Impression of Change
PGIC-PF: PGIC-Physical Functioning
PGIS: Patient Global Impression of Severity
PGIS-PF: PGIS-Physical Functioning
PRO: Patient-reported outcome
PROMIS PF SF: Patient-Reported Outcomes Measurement Information System Physical Function Short Form
SD: Standard deviation
SEM: Standard error of measurement
T2DM: Type 2 diabetes mellitus
